# The prevalence of lynch syndrome (DNA mismatch repair protein deficiency) in patients with primary localized prostate cancer using immunohistochemistry screening

**DOI:** 10.1186/s13053-023-00265-1

**Published:** 2023-10-12

**Authors:** Suguru Oka, Shinji Urakami, Kiichi Hagiwara, Michikata Hayashida, Kazushige Sakaguchi, Yuji Miura, Naoko Inoshita, Masami Arai

**Affiliations:** 1 Department of Urology, Toranomon Hospital, 2-2-2 Toranomon Minato-ku, 105-8470 Tokyo, Japan; 2 Department of Medical Oncology, Toranomon Hospital, 2-2-2 Toranomon Minato-ku, 105-8470 Tokyo, Japan; 3 Department of Pathology, Toranomon Hospital, 2-2-2 Toranomon Minato-ku, 105-8470 Tokyo, Japan; 4 Center for Genetics and Medical Care, Toranomon Hospital, 2-2-2 Toranomon Minato-ku, 105-8470 Tokyo, Japan; 5 Department of Clinical genetics, Graduate School of Medicine, Juntendo University, 2-1-1, Bunkyo-ku, 113-8421 Tokyo, Japan

**Keywords:** Prostate cancer, Lynch syndrome, DNA mismatch repair

## Abstract

**Background:**

Prostate cancer is one of the most heritable human cancers. Lynch syndrome is an autosomal dominant inheritance caused by germline mutations in DNA mismatch repair (MMR) genes, which are also associated with an increased incidence of prostate cancer. However, prostate cancer has not been defined as a Lynch syndrome-associated cancer. The proportion of Lynch syndrome patients in primary prostate cancers is unclear. In this study, we investigated MMR protein loss using universal immunohistochemical screening to determine the prevalence of Lynch syndrome in patients with localized prostate cancer who underwent radical prostatectomy.

**Methods:**

One hundred twenty-nine surgical specimens from radical prostatectomy performed at Toranomon Hospital between 2012 and 2015 were retrospectively tested using universal screening with immunohistochemistry staining for expression of the MMR proteins MLH1, PMS2, MSH2, and MSH6. For all suspected MMR-deficient patients, germline genetic tests focusing on MMR genes were performed.

**Results:**

MMR protein loss was found in only one patient (0.8%) who showed dual MSH2/MSH6 loss. This patient showed a single nucleotide pathogenic germline mutation from c.1129 C to T (p.Gln377*) at exon 7 in the *MSH2* gene. He was diagnosed with a primary prostate cancer at 66 years of age. He had a documented history of Lynch syndrome (Muir–Torre syndrome) with previous colon cancer, sebaceous tumor, and keratoacanthoma as well as subsequent bladder cancer, all of which also showed dual MSH2/MSH6 loss. He also had a strong family history of colorectal and other Lynch syndrome-associated cancers. The pathological stage was pT3aN0M0, and the pathological grade was Gleason 7(4 + 3) with tertiary pattern 5.

**Conclusions:**

In this study, immunohistochemical screening of MMR proteins for Lynch syndrome was performed in a series of prostate cancer cases. The prevalence of Lynch syndrome in localized prostate cancer was 0.8%, which is low compared with other Lynch syndrome-associated cancers.

## Background

Prostate cancer is the most common cancers in men worldwide, with an estimated 1,400,000 cases and 375,000 deaths annually [1]. As of 2018 in Japan, 92,000 people had been diagnosed with the disease, and 12,000 of them died [2].

Risk factors for developing prostate cancer include age, ethnicity, and genetic factors. Prostate cancer is one of the most heritable human cancers. Population-based studies from the Prostate Cancer Database Sweden reported that prostate cancer heritability was estimated to be 57%, which is higher than the rates for almost all other cancers including breast, kidney, and ovarian cancer (31%, 38%, and 39% respectively) [3]. Mutations of DNA damage repair genes including *BRCA2*, *BRCA1* [4, 5], *ATM* [6], and/or mismatch repair (MMR) genes are associated with an increased incidence of prostate cancer [7–9]. Among them, in *BRCA2* mutation carriers, the relative risk of developing prostate cancer by 65 years of age is 7.33-times higher than in non-carriers [10].

Lynch syndrome, which is also known as hereditary non-polyposis colorectal cancer (HNPCC), is an autosomal dominant inheritance caused by germline mutations in MMR genes or loss of MSH2 expression due to *EPCAM* gene deletion. MMR is a necessary component of genome maintenance, and MMR genes include *MLH1, MSH2*, *MSH6*, and *PMS2*. In the revised Bethesda criteria, Lynch syndrome-associated tumors include those of the stomach, ovaries, pancreas, ureter and renal pelvis, biliary tract, and brain. Additionally, sebaceous gland adenomas and keratoacanthomas are indicative of Muir-Torre syndrome, and carcinomas of the small bowel are also considered. [11]. Some reports suggest that men with Lynch syndrome are at a higher risk for prostate cancer. Raymond et al. reported that the risk of prostate cancer in men with Lynch syndrome is estimated to be approximately twice as high as that in the general population [12]. The IMPACT study suggested that carriers of *MSH2* and *MSH6* pathogenic variants have a higher incidence of prostate cancer compared with non-carrier controls, and therefore prostate-specific antigen (PSA) screening may be useful in Lynch syndrome [9]. However, prostate cancer has not been defined as a Lynch syndrome-associated cancer.

Whether prostate cancer in MMR mutation carriers is a more aggressive disease feature has not been studied sufficiently. However, some patients with MMR deficiency progress to castration-resistant metastatic prostate cancer. Recently, pembrolizumab, an anti-PD-1 drug, was approved for efficacy in patients with high frequency of microsatellite instability (MSI-H) and DNA mismatch repair deficiency (dMMR) solid tumors. Using this drug to treat dMMR prostate cancers may significantly change the prognosis of metastatic castration-resistance prostate cancer for the better in the future [13]. Therefore, studying the prevalence of Lynch syndrome in prostate cancers is important.

A study on the prevalence of germline DNA repair gene mutations in men with metastatic prostate cancer found *BRCA2* mutations in 5.3% and dMMR in 0.7% of these patients [14]. However, the proportion of Lynch syndrome patients in primary prostate cancers is still unclear. In this study, we investigated universal MMR protein immunohistochemical screening to determine the prevalence of Lynch syndrome in patients with localized prostate cancer who underwent radical prostatectomy.

## Methods

### Ethical considerations

The Institutional Review Board at Toranomon hospital approved this study (reception No. 2345) and informed consent was obtained in the form of opt-out on the website. Each patient’s clinical data were confirmed from their medical records.

### Patients

From January 1, 2012 to December 31, 2015, there were 129 surgical specimens from radical prostatectomy performed at Toranomon Hospital, Tokyo, Japan. The tumor stage was determined in accordance with the American Joint Committee on Cancer Staging Manual, 8th edition [15], and the Gleason score was assigned in accordance with the 2014 International Society of Urological Pathology consensus [16].

### Immunohistochemistry for DNA mismatch repair proteins

The specimens were retrospectively analyzed using universal screening with immunohistochemistry (IHC) to identify the MMR proteins MLH1, PMS2, MSH2, and MSH6. To achieve high standardization in IHC analysis, we fabricated a tissue microarray comprising both tumor and control tissues. These were extracted as cores measuring 3 micrometers in thickness and 3 millimeters in diameter from each paraffin-embedded block. We employed the standard avidin-biotin-peroxidase complex method for the analysis, utilizing an automated immunostainer (BenchMark XT; Ventana Medical Systems, Tucson, AZ, USA), and executed the procedure independently of any clinical criteria. The antibodies applied were as follows: MLH1 (M3640, DAKO, Glostrup, Denmark; dilution 1:50), MSH2 (#2017, Cell Signaling Technology Japan, Tokyo, Japan; dilution 1:200), MSH6 (M3646, DAKO, Glostrup, Denmark; dilution 1:50), and PMS2 (M3647, DAKO; dilution 1:40). MMR deficiency was defined as the loss of protein expression by IHC. If the tumor showed total absence of nuclear staining with the adjacent normal tissue showing normal nuclear staining, the specimen was considered to be a deficient MMR tumor. An experienced pathologist (NI) who was blinded to clinical variables and outcome information reviewed all the IHC slides.

### Genetic testing

For patients suspected of having MMR deficiency based on IHC, we recommended genetic counseling for Lynch syndrome at our collaborating institutions. During these counseling sessions, medical professionals provided a comprehensive explanation of the implications of Lynch syndrome and the importance of genetic testing. If the patient expressed a desire for genetic testing, a germline genetic analysis focusing on MMR genes was carried out. The genetic variant analysis was outsourced to FALCO (Kyoto, Japan). The initial screening was conducted using next-generation sequencing (NGS), and if a genetic variant was identified, its presence was further confirmed using Sanger sequencing.

## Results

### Patient characteristics

Only patients with localized prostate cancer underwent surgical treatment. Patient characteristics are shown in Table 1. There were 36 (28%) men with Gleason scores 8 to 10 (Grade Group 4–5), which was classified as high risk or higher. The four patients who underwent hormone therapy before surgery and did not evaluate their Gleason scores. A detailed analysis of tumor profiles was conducted on our study population. Table 2 shows data for 20 patients (15.5%) diagnosed with various types of tumors. Among these, five patients (3.8%) had colorectal cancer, and another five exhibited Lynch syndrome-associated tumors without colorectal cancer. Only case 1 met the revised Bethesda criteria for Lynch syndrome testing. Moreover, the loss of expression for at least one of the four MMR proteins was observed in only one of the 129 patients (0.8%), which was also case 1.

**Table 1:** Diagnostic characteristics and pathological findings of all patients

Age at diagnosis (years) [median (range)]		68 (52–79)
Initial PSA (ng/mL) [median (range)]		8.4 (3.6–75.0)
Grade group [n (%)]	unknown	4 (3%)
	1	11 (9%)
	2	37 (29%)
	3	41 (32%)
	4	10 (8%)
	5	26 (20%)
pT [n (%)]	2	72 (56%)
	3a	34 (26%)
	3b	23 (18%)
	4	0 (0%)
pN [n (%)]	0	127 (98%)
	1	2 (2%)

**Table 2:** Characteristics of patients with a history of tumors

Case	Colorectal cancer (age at diagnosis))	Lynch syndrome-associated tumors	Other tumors
1	55	Keratoacanthoma	Bladder Cancer
2	58		Lung Cancer
3	67		
4	71		
5	75		
6		Gallbladder Cancer	Kidney Cancer
7		Stomach Cancer, Renal Pelvis Cancer	Pharyngeal Cancer
8–11		Stomach Cancer	
12		Cholangiocarcinoma	
13		Pancreatic Cancer	
14			Thyroid Tumor
15–17			Esophageal Cancer
18			Lung Cancer
19			Bladder Cancer
20			Pituitary Tumor

#### Case summary

**Fig. 1:**
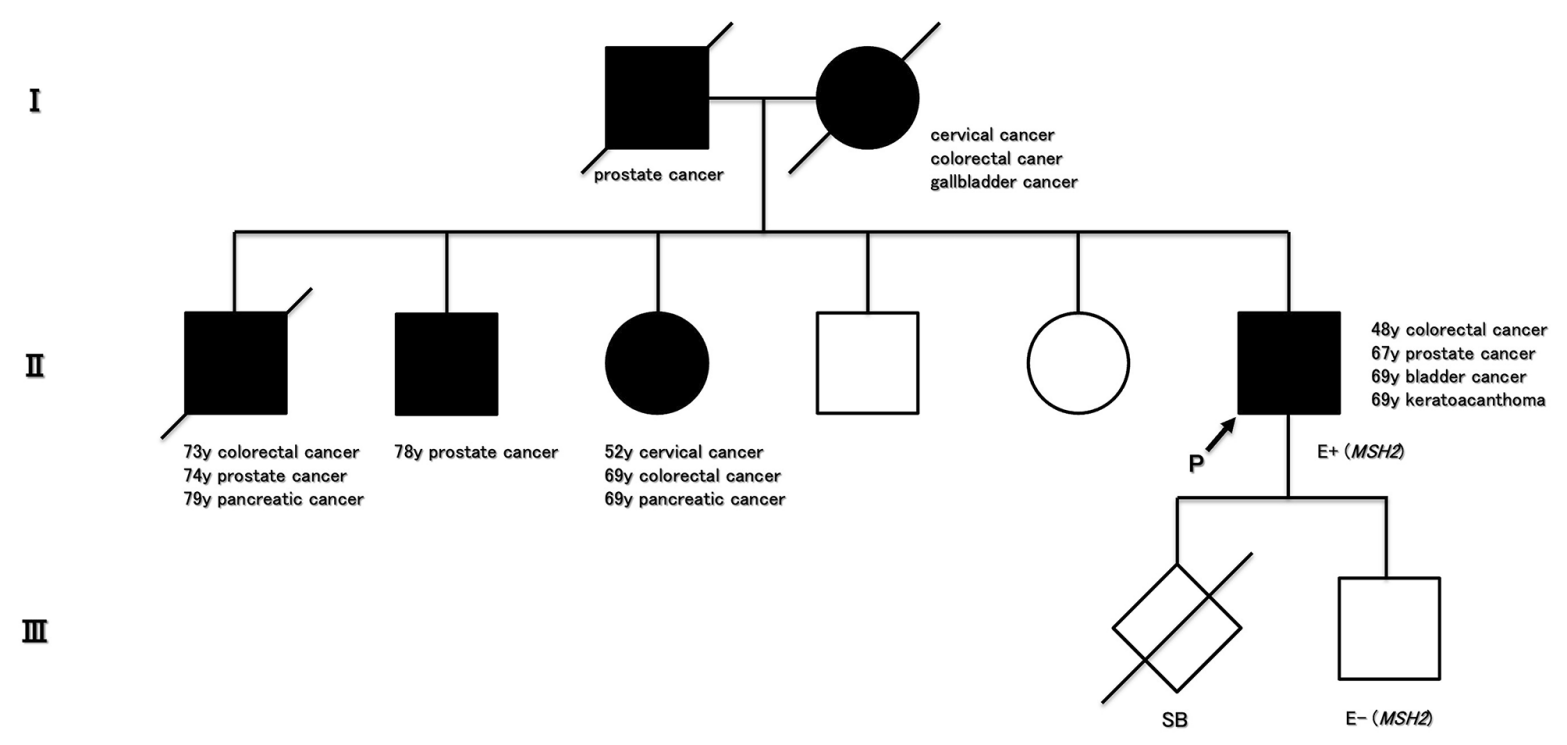
Family pedigree: In the pedigree chart, ‘P’ denotes the proband, ‘E+(*MSH2*)’ indicates *MSH2* genetic testing positive, ‘E-(*MSH2*)’ indicates *MSH2* genetic testing negative, and ‘SB’ represents stillbirth

Only one patient, identified as case 1, showed loss of MMR expression, specifically dual MSH2 and MSH6 loss. He was diagnosed with primary prostate cancer at 66 years of age. He had a documented history of Lynch syndrome with previous colon cancer (55 years old), sebaceous tumor and keratoacanthoma (68 years old), and subsequent bladder cancer (68 and 70 years old). Additionally, he had a strong family history of colorectal and other Lynch syndrome-associated cancers (Fig. 1) [17]. Based on these findings, we suspected the diagnosis of Lynch syndrome and performed genetic testing to confirm it, and then we identified a pathogenic germline mutation in exon 7 of MSH2 (c.1129 C > T, p.Gln377Ter), and the patient was diagnosed with Lynch syndrome. Finally, he was diagnosed with Muir–Torre syndrome because he was genetically diagnosed Lynch syndrome with keratoacanthoma [18].

#### Prostate cancer

At 66 years of age, he had a PSA level of 5.82 ng/mL on a routine evaluation. His physical examination results were normal, and the digital rectal examination revealed a slightly enlarged prostate. Prostatic biopsy revealed a Gleason score of 8(4 + 4) and adenocarcinoma in six of 14 specimens. Chest, abdominal, and pelvic computed tomography scans and bone scan results demonstrated no evidence of nodal or visceral metastasis.

**Fig. 2:**
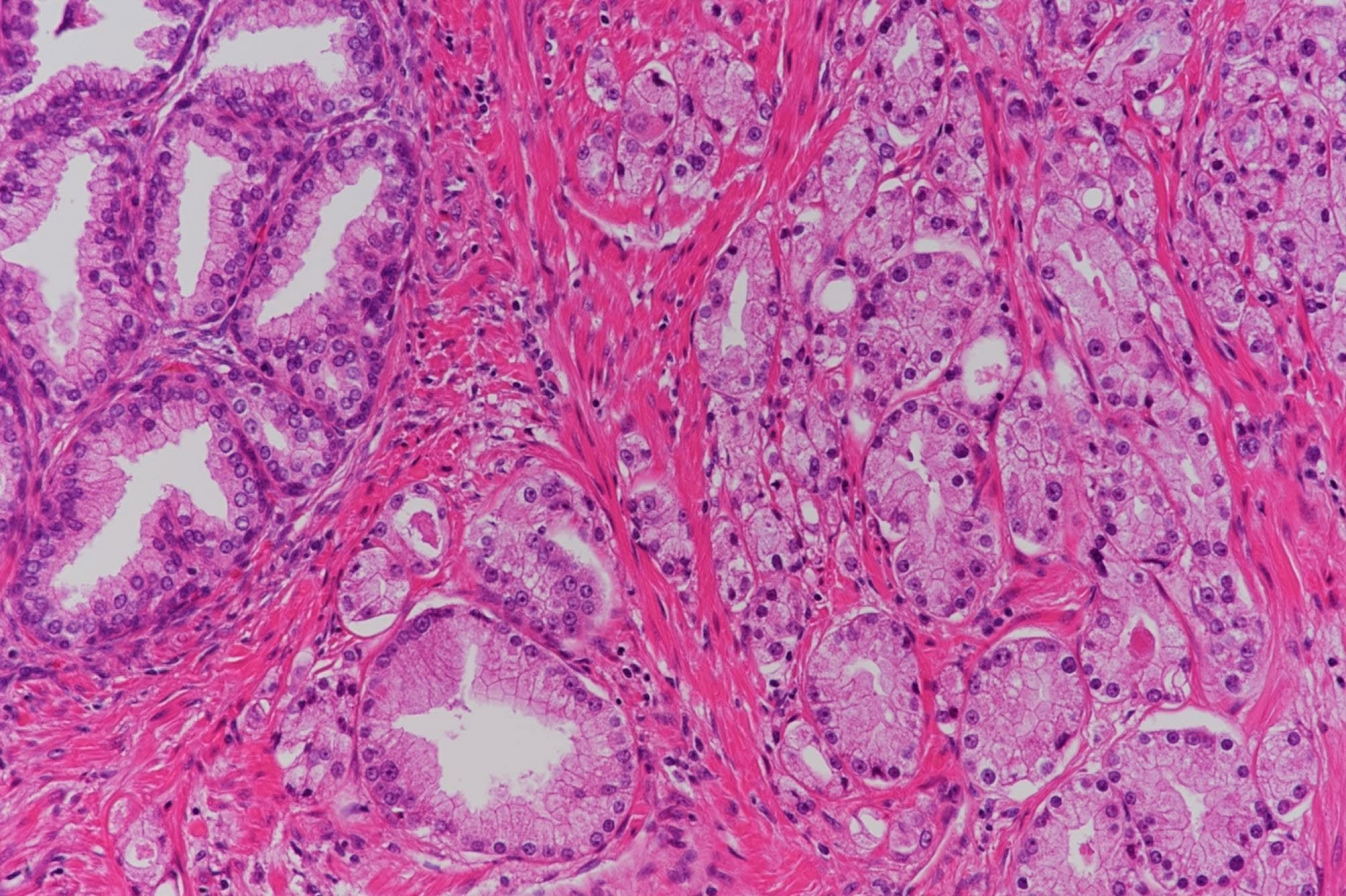
Initial serum PSA 5.82 ng/mL, adenocarcinoma, GS 4 + 3 with tertiary 5, pT3aN0, EPE1, RM1, ly0, v1, pn1. H&E × 40

**Fig. 3:**
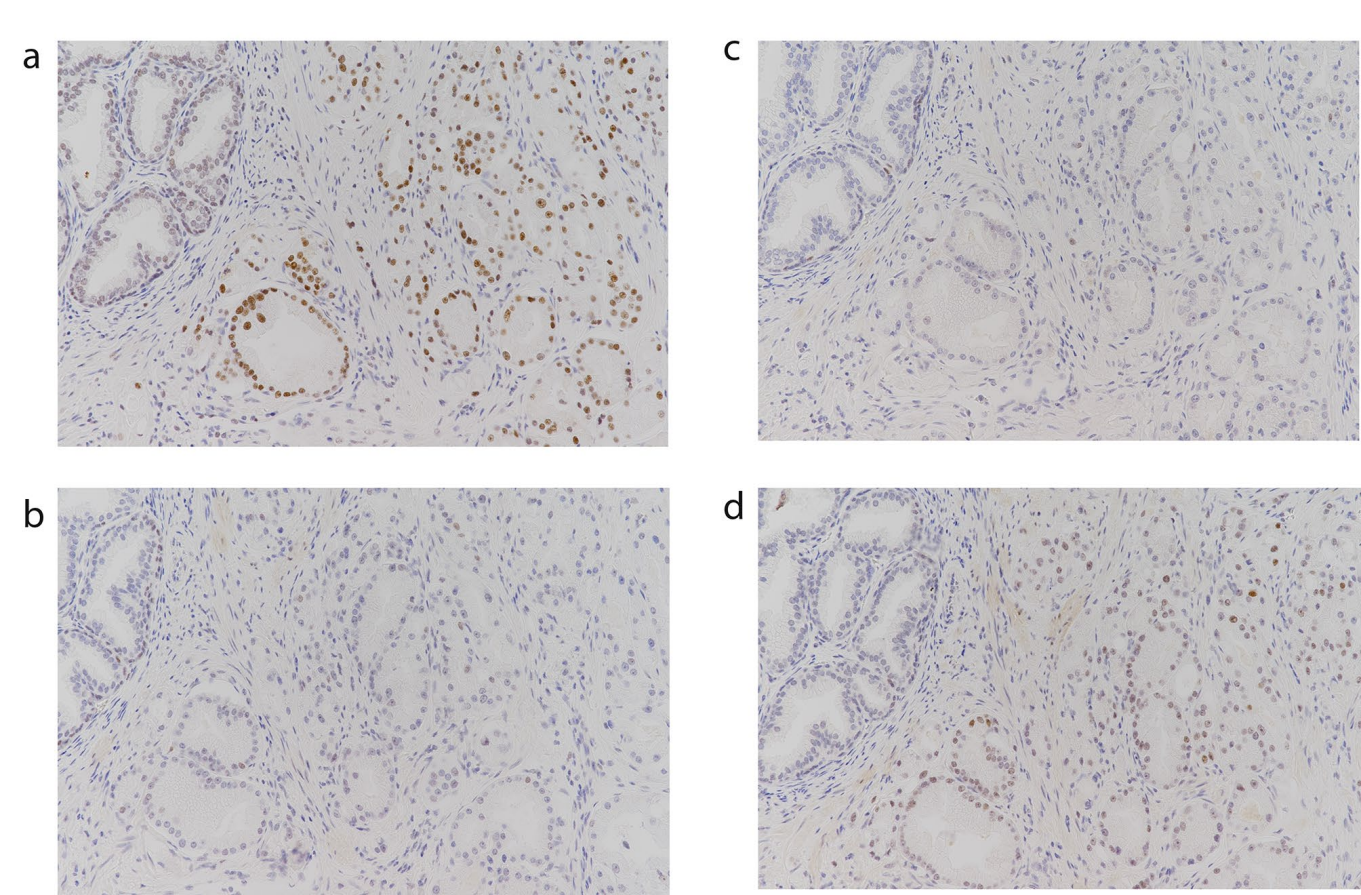
Immunohistochemistry staining images for the four MMR proteins in prostate cancer in Case 1. (a) MLH1, (b) MSH2, (c) MSH6, (d) PMS2. Loss of nuclear staining in MSH2 and MSH6 are shown. IHC × 40

He underwent a radical retropubic prostatectomy and the final pathology revealed pT3aN0M0, a Gleason score 7(4 + 3) with tertiary pattern 5 adenocarcinoma (Fig. 2) involving positive capsule penetration and a positive surgical margin. Pathological lymph node metastasis was negative. IHC staining of prostate cancer for MMR identified dual loss of MSH2 and MSH6 (Fig. 3). For 5 years after surgery, his PSA level was undetectable.

#### Bladder cancer, colorectal cancer, and keratoacanthoma

At 68 years of age, a bladder tumor was discovered during routine transabdominal ultrasound examination in this case. Cystoscopy revealed that there were three pedunculated papillary lesions that were up to 2 cm in size, occupying the right bladder wall. Transurethral resection was performed without complications. The final pathology revealed low-grade pTa. IHC staining of the patient’s bladder cancer revealed four MMR proteins, with dual loss of MSH2/MSH6. Bladder cancer recurred 2 years later, and the patient underwent surgery again.

Immunohistochemical staining showed double loss of MSH2 and MSH6 using immunohistochemical staining for colorectal cancer, keratoacanthoma, and prostate and bladder cancer.

## Discussion

Here, we described a retrospective universal screening protocol that was conducted at our institution in which all prostatectomy samples that showed prostate cancer were screened for MLH1, PMS2, MSH2, and MSH6 deficiencies using IHC. Loss of expression in at least one of the four MMR proteins was found in one of 129 patients (0.8%). In a report from Japan, Kagawa et al. reported that 337 patients who underwent prostatectomy for prostate cancer were screened for MMR protein loss using IHC, and four patients showed loss of MMR proteins (1.2%) [19]. However, their study found no immunohistochemical defects in the MMR protein with germline mutations. These may all be acquired somatic mutations, and they have not been genetically proven to be Lynch syndrome. However, our study identified one patient with MMR protein defects using IHC who subsequently had a germline mutation that was identified using genetic testing (0.8%). Our study is the first report that estimates the prevalence of Lynch syndrome among Asian prostate cancers. For other races, Abida et al. reported the prevalence of MSI-H/dMMR prostate cancer. They tested 1033 men, and 32 (3.1%) had MSI-H/dMMR prostate cancer, while seven patients (0.6%) had germline mutations [20]. These results including ours indicated that the prevalence of a germline mutation in MMR was less than 1%, regardless of racial differences. Therefore, our study evaluating the suitability of universal MMR IHC screening for prostate cancers showed that it was not necessary to perform universal IHC screening for all patients who underwent radical prostatectomy.

In our study, the patient with Lynch syndrome was diagnosed with Muir–Torre syndrome. Muir–Torre syndrome is a rare autosomal dominant disease characterized by at least one sebaceous gland tumor and at least one simultaneous or heterochronic visceral tumor, and it is caused by germline mutations in the MMR gene. Muir–Torre syndrome is considered to be a phenotypic variant of Lynch syndrome. Approximately 9% of Lynch syndrome patients are considered to have Muir–Torre syndrome [21]. Most patients with Muir–Torre syndrome carry a germline mutation in *MSH2*. The Mayo Muir–Torre syndrome score is used to determine the likelihood of having Muir–Torre syndrome [22]. If the score is 2 or higher, genetic testing is recommended. We strongly suspected Muir–Torre Syndrome because our patient had a score of 4 points. After providing genetic counseling to the patient, he underwent genetic testing. The results showed pathogenic germline mutation of *MSH2* in this patient, who was also deficient in MSH2 and MSH6 proteins, as shown using IHC staining.

There are no reports on the prognosis of prostate cancer with MMR mutations, but there are several reports on the clinical and pathologic features. Antonarakis et al. reported that 75% of metastatic prostate cancers with mutations in the MMR gene were high-grade with a Gleason score of 9–10. However, they were sensitive to standard and novel hormone therapies. Additionally, most patients had MSH6 (46%; 6/13) or MSH2 (23%; 3/13) mutations [23]. Another report on MSH2-deficient prostate cancer found that 8% (7/91) of adenocarcinomas with a Gleason score of 9–10 are MSH2-deficient compared to 0.4% (5/1,042) of adenocarcinomas with other scores [24]. Because most patients with Muir–Torre syndrome are MSH2-deficient and prostate cancers with MSH2 deficiency tend to have higher Gleason scores, early detection and treatment are important for prostate cancer patients with Muir–Torre syndrome. Our patient with Muir–Torre syndrome had a locally advanced cancer stage with tertiary pattern 5, which was thought to be highly malignant, but there has been no recurrence for the subsequent 5 years due to relatively early prostatectomy.

The study had several limitations. First, this is a single-center, retrospective study with a small number of cases. A larger number of cases would allow for more accurate prevalence estimates. Second, we employed only IHC as the universal screening method for prostate cancer. This may lead to underestimation of Lynch syndrome frequency. There are no studies on the sensitivity and specificity of IHC in prostate cancer and other Lynch syndrome-related tumors. In colorectal cancer, sensitivity and specificity with IHC are approximately 88.9–92.4% and 87.8–87.9% respectively [25]. In contrast, the sensitivity of MSI was slightly higher at 92.9%, but the number of cases that were ineligible for testing was higher for MSI (14%) compared to IHC (0.3%). A high concordance rate of 99.1% was observed between IHC and MSI in testable cases. This suggests that IHC may be more practical for universal screening due to the high proportion of untestable cases with MSI and the high concordance rate in testable cases. We chose IHC because of its simplicity and cost-effectiveness.

## Conclusions

This study examined all four MMR proteins using IHC screening in primary prostate cancers. The prevalence of MMR mutations in patients with localized prostate cancer was 0.8%, which was a lower frequency than in other Lynch syndrome-associated cancers.

## Data Availability

The data that support the findings of this study are available from Suguru Oka but restrictions apply to the availability of these data, which were used under license for the current study, and so are not publicly available. Data are however available from the authors upon reasonable request and with permission of the Institutional Review Board at Toranomon hospital.

## Abbreviations

MMR: DNA mismatch repair
HNPCC: Hereditary non-polyposis colorectal cancer
PSA: Prostate-specific antigen
MSI-H: High frequency of microsatellite instability
dMMR: DNA mismatch repair deficiency
IHC: Immunohistochemistry
NGS: Next-generation sequencing
